# Computational Approaches Integrated in a Digital Ecosystem Platform for a Rare Disease

**DOI:** 10.3389/fmmed.2022.827340

**Published:** 2022-02-22

**Authors:** Anna Visibelli, Vittoria Cicaloni, Ottavia Spiga, Annalisa Santucci

**Affiliations:** ^1^ Department of Biotechnology, Chemistry and Pharmacy, University of Siena, Siena, Italy; ^2^ Toscana Life Sciences Foundation, Siena, Italy; ^3^ Competence Center ARTES 4.0, Siena, Italy; ^4^ SienabioACTIVE—SbA, Siena, Italy

**Keywords:** alkaptonuria, rare disease, machine learning, precision medicine, data analysis, bioinformatics

## Abstract

Alkaptonuria (AKU) is an ultra-rare autosomal recessive disease caused by a mutation in the homogentisate 1,2-dioxygenase gene. One of the main obstacles in studying AKU and other ultra-rare diseases, is the lack of a standardized methodology to assess disease severity or response to treatment. Based on that, a multi-purpose digital platform, called ApreciseKUre, was implemented to facilitate data collection, integration and analysis for patients affected by AKU. It includes genetic, biochemical, histopathological, clinical, therapeutic resources and Quality of Life (QoL) scores that can be shared among registered researchers and clinicians to create a Precision Medicine Ecosystem. The combination of machine learning applications to analyse and re-interpret data available in the ApreciseKUre clearly indicated the potential direct benefits to achieve patients’ stratification and the consequent tailoring of care and treatments to a specific subgroup of patients. In order to generate a comprehensive patient profile, computational modeling and database construction support the identification of potential new biomarkers, paving the way for more personalized therapy to maximize the benefit-risk ratio. In this work, different Machine Learning implemented approaches were described:

• Predictive model for the estimation of oxidative status trend of each AKU patient based on different biochemical predictors (Cicaloni et al., 2019).
• Prediction of QoL scores based on clinical AKU patients’ clinical data to perform patients’stratification (Spiga et al., 2020).
• A tool able to investigate the most suitable treatment in accordance with AKU patients’ QoL scores (Spiga et al., 2021a).
• The comparison of different algorithms to explore the phenotype–genotype relationships unknown in AKU so far (Spiga et al., 2021b).

We also implemented an ApreciseKUre plugin, called AKUImg (Rossi et al., 2020), dedicated to the storage and analysis of AKU histopathological slides, where images can be shared to extend the AKU knowledge network. The outcomes of these predictions highlight the necessity of development databases for rare diseases like ApreciseKUre. We believe this is not limited to the study of AKU, but it could be applied to other rare diseases, allowing data management, analysis, and interpretation.

## Introduction

Although evidence-based medicine (EBM) has been the main guide for medical treatment over the last decades, this approach does not consider the individual molecular characteristics of the patients, which are of great importance for the efficacy and safety of therapies. Indeed, the decision-making process in medical practice that considers only the most reliable scientific information combined with the individual expertise of the clinician (Bereczki, 2012), cannot be generalized for all patients. It is well known that not all people respond to therapies and drugs in the same way (Hafen et al., 2014; Lehrach, 2015; Roden, 2015) for their differences in genomic, epigenomic and metabolomic profile (Leyens et al., 2014) and other several factors including diet, comorbidities, age and weight (Haga, 2017). In fact, it is possible that patients do not improve their condition after taking the drugs recognized as the “best” for that pathology, or even suffer from more serious complications due to the accompanying side effects such as adverse drug reactions (ADRs). To maximize the benefit/risk ratio, pharmaceutical interventions and dosage should be specifically tailored for individual patients on their disease risk and expected response.

To address this problem, a new approach called Precision Medicine (PM) has become a reality in recent years. This recent technique focuses on different individual parameters, such as genes variability, environment, lifestyle, and various biological markers (www.nih.gov/precision-medicine-initiative-cohortprogram) for the prevention and treatment of diseases. Biomarkers, for example, are biological indicators that could have a specific molecular, anatomic, physiologic, or biochemical character, which can be accurately detected and evaluated (Biomarkers Definition Working Group, 2001). They play a key role as indicators of an ordinary or pathogenic biological process, having a specific physical characteristic or biological change produced. Thanks to PM it is possible primarily to promote research and understanding a wide range of diseases, but also to identify the causes of the different responses to drugs commonly used to treat different patients. Patients can be “stratified” (Laifenfeld et al., 2012) according to their susceptibility to a particular disease or their response to a specific treatment. The PM approach is already profitably applied in various health areas such as oncology, cardiology, nutrition, and in particular rare diseases (Schee Genannt Halfmann et al., 2017; Trusheim et al., 2011).

## Precision Medicine in an Ultra-rare Disease

While the PM has focused on large amounts of data to study more common diseases, the data obtained from rare diseases are often limited and sparse. This lack of information makes the ability to collect, integrate and analyze data an extremely difficult but necessary effort. Therefore, to overcome this obstacle, PM in rare diseases focuses on creating patients’ registries, leveraging the largest amounts of data available to discover potential connections and including patients as active partners in this research (Trusheim et al., 2011). A process of data harmonization in rare disease registries allows to conduct clinical studies to understand the complexity of diseases, allowing a more accurate classification based on their genetic characteristics (Ogino et al., 2012).

An obstacle in the creation of such registers is that they are often created at the national or local level, to map rare diseases in certain areas and to gather information on their incidence and prevalence in those selected areas. Data for such disease registries are mostly obtained on a voluntary basis, observational studies, and clinical data. It would be desirable that such registers could be also strengthened by expanding data thanks to the implementation of PM in health systems across the EU (Schee Genannt Halfmann et al., 2017).

In this review, we focused our attention on the application of Artificial Intelligence techniques to analyze and re-interpret data on Alkaptonuria (AKU), an ultra-rare disease characterized by no apparent genotype-phenotype relationship and no prognosis. Our overall goal was to advance research on rare orphan AKU disease towards a PM approach that addresses disease complexity while considering individual variability.

From a PM perspective, a digital platform dedicated to AKU called ApreciseKUre was created (www.bio.unisi.it/aprecisekure/; www.bio.unisi.it/aku-db/), containing data collected from all over the world from different information levels. The ApreciseKUre platform was not created as a simple registry, but rather as a Precision Medicine Ecosystem (PME) in which genetic, biochemical, and clinical resources are shared between researchers, clinicians, and patients (Aronson and Rehm, 2015) in order to promote a better understanding of the pathophysiological mechanisms of AKU and related comorbidities.

## Alkaptonuria

AKU in an ultra-rare autosomal recessive disease caused by the mutations of the Homogentisate 1,2- dioxygenase (HGD) gene which leads to a deficiency of the HGD enzyme (Ascher et al., 2019; La Du et al., 1958) producing accumulation of the unprocessed toxic catabolite homogentisic acid (HGA), especially in connective tissues. AKU was the first disorder to conform with the principles of Mendelian recessive inheritance (Garrod, 1908) with an estimated incidence of 1 case in 250.000–1.000.000 births in most ethnic groups (Phornphutkul et al., 2002) and around 1300 patients around the world (Zatkova et al., 2020; Ascher et al., 2019). At a structural level, the active form of the HGD enzyme is a complex hexamer (Titus et al., 2000) with low tolerance to mutations, including missense variants, which can damage protein folding stability and alter HGA accumulation (Nemethova et al., 2016). While the excess HGA is mostly eliminated through urine, the remaining portion contributes to the production of an ocronotic pigment deposited in cartilage (Milch, 1961; Braconi et al., 2015; Bernardini et al., 2019; Bernini et al., 2021; Braconi et al., 2020), which contributes in arthropathy early onset, responsible for reducing patients’ quality of life and causing severe pain and deficit in locomotion (Milch, 1961; Braconi et al., 2015; Spiga et al., 2020). Oxidative stress and chronic inflammation are also triggered by the HGA accumulation (Braconi et al., 2015; Braconi et al., 2010; Braconi et al., 2011; Millucci et al., 2014) in different organs, making AKU a complex multisystemic disease. Lately, AKU has been classified as a secondary amyloidosis (Millucci et al., 2014; Millucci et al., 2012; Millucci et al., 2015), characterized by the deposition of serum amyloid A (SAA) fibers, a circulating protein produced at high levels in chronic inflammation, making SAA a sensitive biomarker (Gabay and Kushner, 1999), confirmed by elevated SAA plasma levels also in AKU patients (Millucci et al., 2014, Millucci et al., 2012, Millucci et al., 2015, Braconi et al., 2016, Braconi et al., 2018). Moreover, both ochronotic pigment and SAA-amyloid share the same location in human cartilage and other tissues (Millucci et al., 2012). In addition to SAA, another marker of chronic inflammation is chitotriosidase (CHIT1) (Cho et al., 2014). CHIT1 can be considered a biomarker of AKU as it is linked to other diseases such as sarcoidosis, rheumatoid arthritis, and ankylosing spondylitis (Cho et al., 2014; Braconi et al., 2018). In AKU, in addition to inflammation, patients also suffer from significant oxidative stress caused by high systemic levels of HGA showing interesting similarities with other rheumatic diseases (Braconi et al., 2016). In this context, Protein Thiolation index (PTI) interestingly denotes and summarizes the oxidative status of AKU patients, as revealed by ApreciseKUre tools and experimentally confirmed (Cicaloni et al., 2019). The lack of a standardized methodology to assess disease severity and response to treatment, which is highly variable from individual to individual, appears to be a critical issue in AKU (Vilboux et al., 2009; Ranganath and Cox, 2011; Ascher et al., 2019) requiring a reliable way to monitor patients’ clinical conditions and overall health status. A way to help to identify health needs and to evaluate the impact of the disease is represented by the measure of Quality of Life (QoL) (Braconi et al., 2018) whose correlation with the clinical data deposited in the ApreciseKUre database may help to effectively face AKU complexity (Spiga et al., 2020).

## ApreciseKUre Digital Ecosystem Platform

**FIGURE 1:**
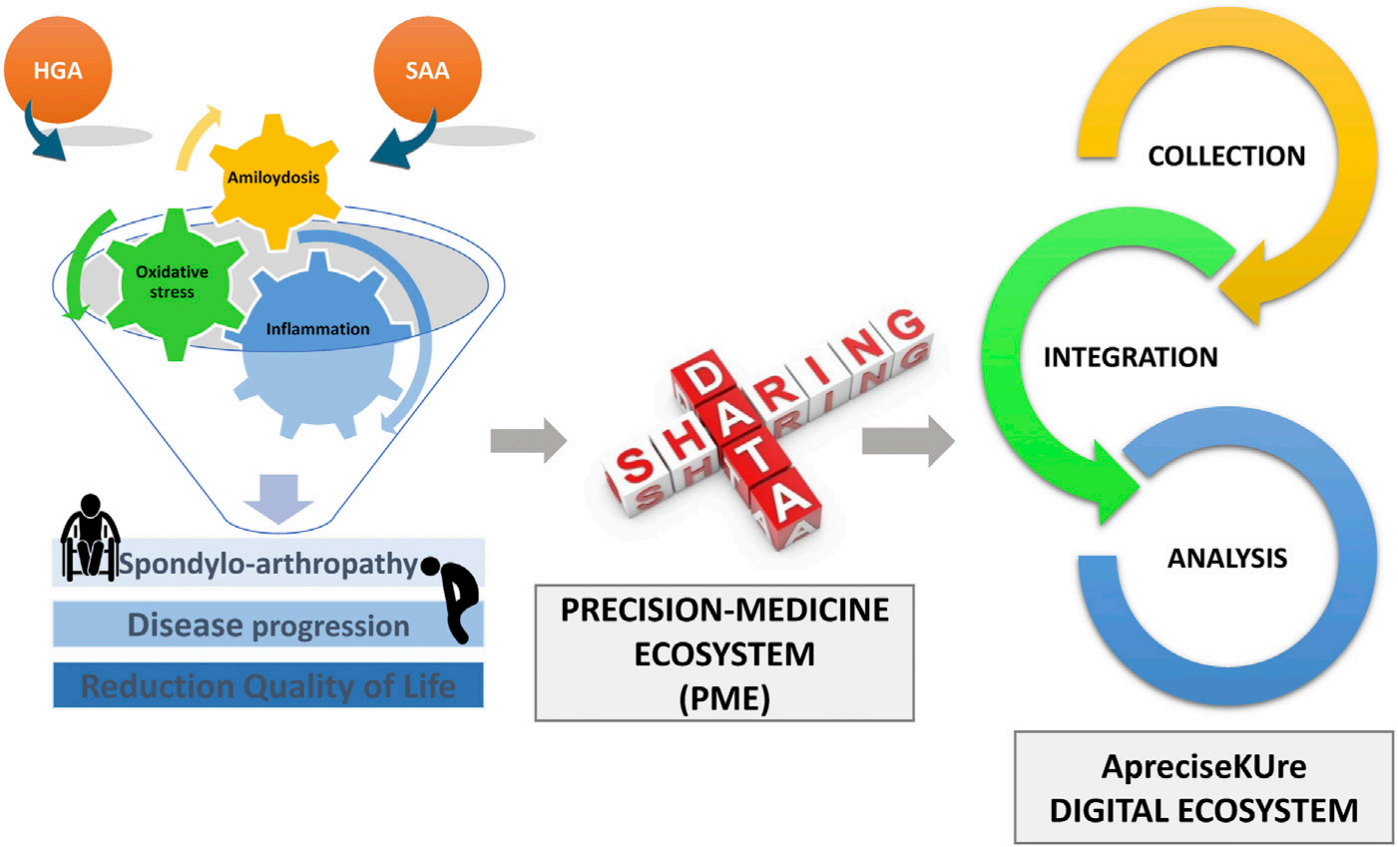
ApreciseKUre digital ecosystem. AKU dedicated Precision Medicine Ecosystem (PME).

The aim was of ApreciseKUre is to develop an AKU-PME in which patient-derived information (QoL), clinician-derived information, and mutational analysis can be collected, integrated and shared between scientists, clinicians and patients (Spiga et al., 2017 and Spiga et al., 2018), to build a worldwide easily consultable reference point for AKU (Figure 1).

**FIGURE 2:**
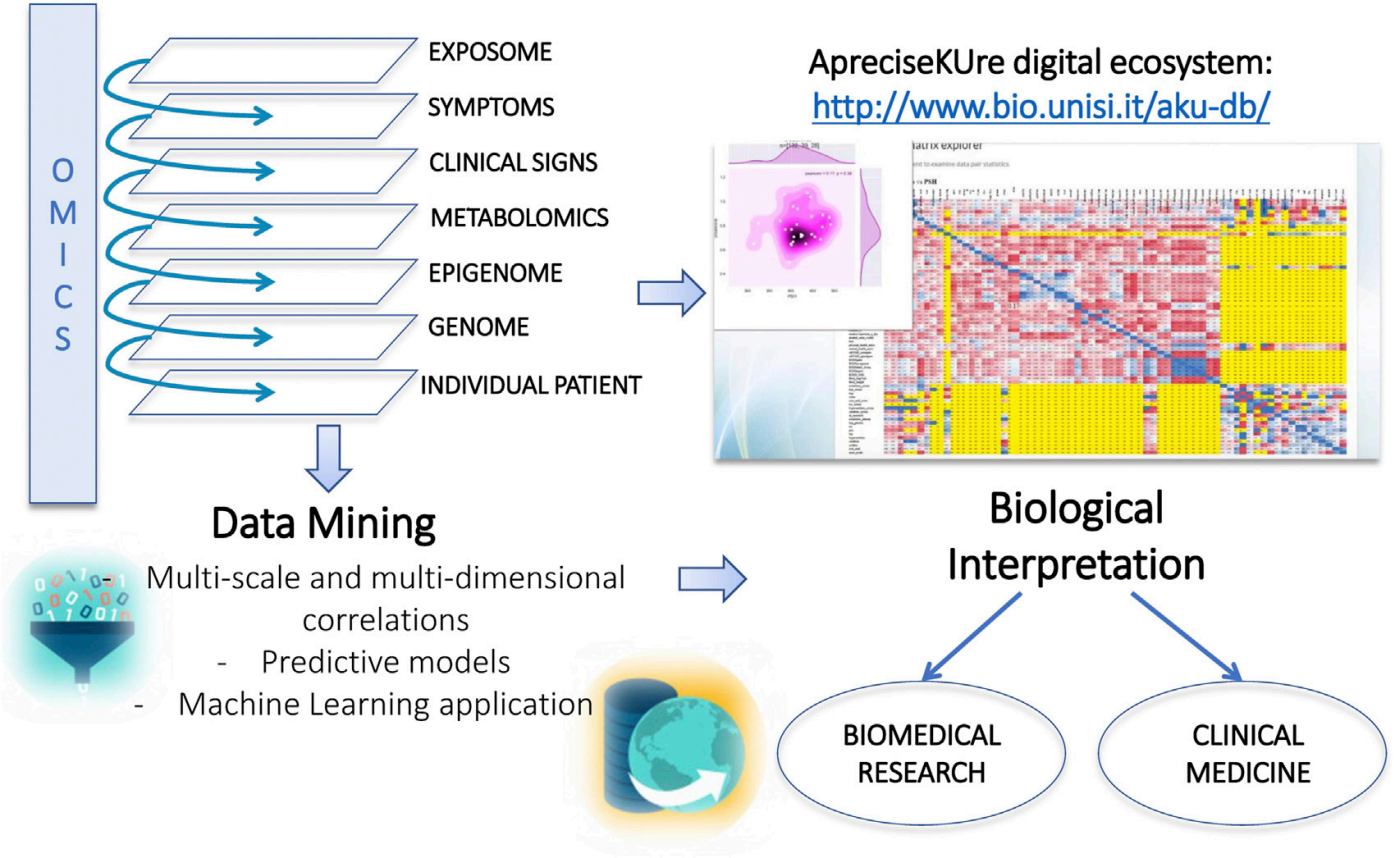
ApreciseKUre structure. Data stratification into various -omics levels and different types of data mining that support biomedical research and clinical medicine.

In detail, AKU patients’ data have been collected and divided into different levels such as genetic, protein, biochemical, histopathologic, clinical, lifestyle and habitual, as shown in Figure 2.

Currently, ApreciseKUre (Spiga et al., 2021a and Spiga et al., 2021b) incorporates data of over 210 subjects with AKU, 119 more than its original version (Cicaloni et al., 2016; Spiga et al., 2017; Spiga et al., 2018) which is an exceptional result considering the rarity of AKU. The total number of fields making up each record is 110, with 82 numeric attributes and 8 Booleans; the remaining fields are categorical values (for the complete list see supplementary material by Spiga et al., 2021a and Spiga et al., 2021b).

**FIGURE 3:**
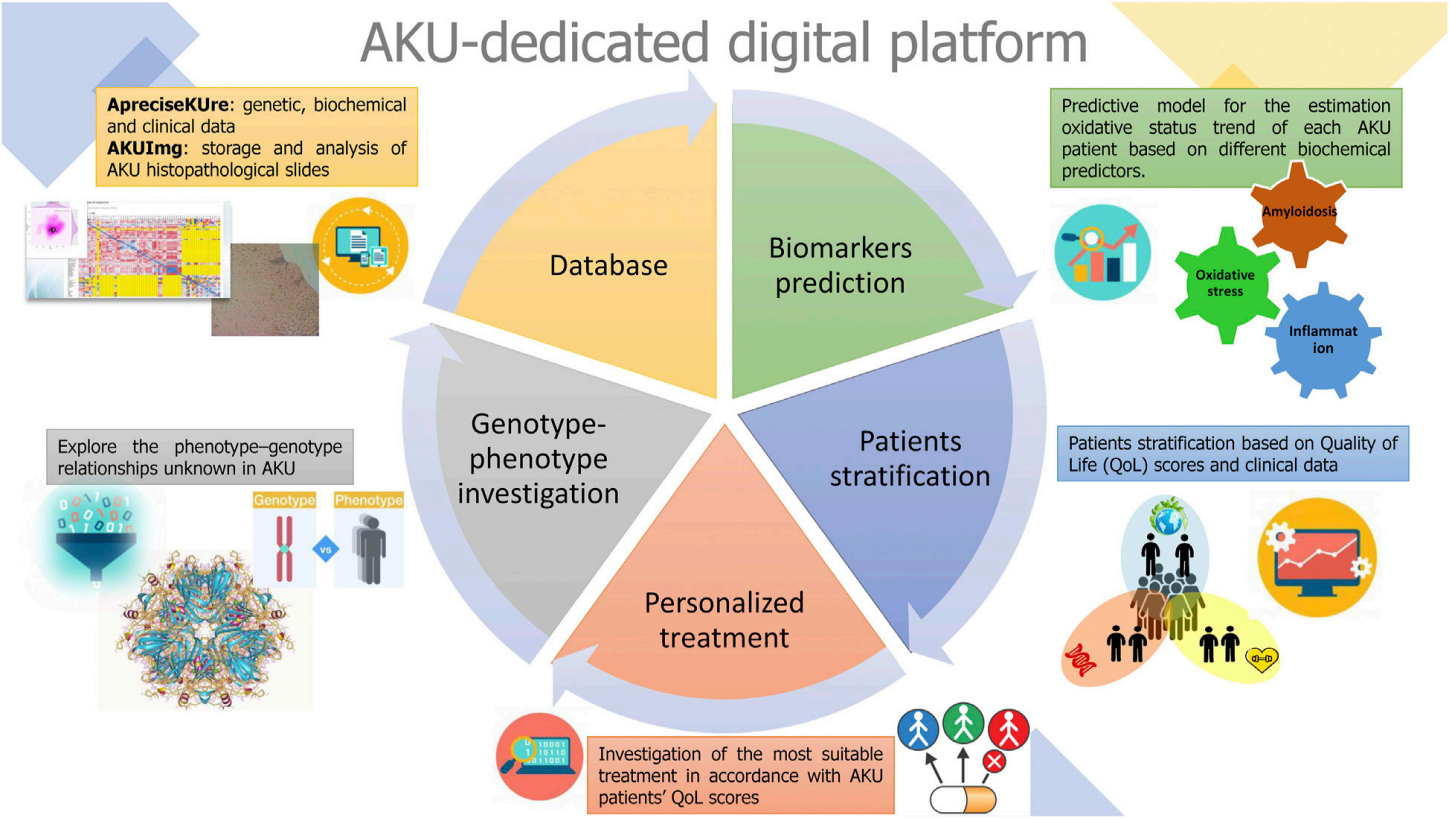
Data mining techniques. All the outcomes derived from statistical and computational approaches included in ApreciseKUre are displayed.

Different data mining techniques were implemented to discover potential biomarkers, opening new opportunities to match therapy to patients, possibly single therapy to a single group of patients, thus leading to a more personalized medicine for maximizing the benefit to risk ratio. The outcomes obtained from these models could be useful not only to advance the treatment of AKU, but also to serve as a model for other rare diseases. In Figure 3, all the data analysis techniques are summarized, ranging from more common statistical data mining to deeper ML models.

### Data Analysis by a Refreshable Correlation Matrix

The first analytical method developed is based on a statistical analysis (Pearson correlation) in which numerical data included in ApreciseKUre are correlated with the consequent creation of a refreshable correlation matrix. The modeling correlations offer significant support for early diagnosis, monitoring and treatment in AKU by revealing that some clinically used biomarkers may not be suitable in AKU.

One of the most interesting results obtained is the inverse correlation between CystatinC (CysC) and Cathepsin D (CatD). CysC is a marker for monitoring renal function: if the glomerular filtration rate (GFR) decreases, blood levels of CysC increase (Randers et al., 1998; Croda-Todd et al., 2007) indicating dysfunctionality. Levels of CatD, a protease capable of degrading proteins such as CysC (Lenarčič et al., 1991), are particularly elevated in rheumatic diseases (Khalkhali-Ellis and Hendrix Mary, 2014) such as AKU. Ochronotic manifestations in AKU gradually lead to kidney stones and nephrolithiasis (Faria et al., 2012). Even though patients with AKU often suffer from renal dysfunction, a subset of AKU patients showed high values of CatD while CysC levels did not increase (Braconi et al., 2018). Starting from a statistical observation, it was possible to biologically suggest that CysC might not be a suitable marker to measure GFR in AKU, since overexpression of CatD in AKU might lead to degradation of CysC, making it no longer detectable.

This first data-mining approach revealed the amount of hidden information which can be extrapolated from computational models, in order to acquire a deeper knowledge of the AKU and to identify prognostic biomarkers that can be exploited for a reliable clinical monitoring. In addition, given the chronic nature of AKU, clinical monitoring of patients’ health status becomes necessary as well as the implementation of a correlation system capable of comparing biomarkers at different times with follow-up studies.

### Predictive Model for the Estimation Oxidative Status

After this preliminary model, a prognostic method based on linear regression able to investigate oxidative stress status of AKU patients, starting from easily measurable clinical parameters (Cicaloni et al., 2019) was integrated in ApreciseKUre. This predictive system could help clinicians to easily monitor the oxidative stress evolution in single patients, with the consequent most appropriate antioxidant treatment prescription for each of them. It has already emerged from the correlation matrix that PTI is a reliable biomarker to monitor oxidative stress in AKU (Giustarini et al., 2017). A linear regression model was then implemented, revealing the most influential biomarkers for PTI prediction, and consequently, for oxidative stress estimation. Such biomarkers are parameters easily measured in AKU clinical analysis and they are related to inflammation, amyloidosis, and lifestyle. They are Body Mass Index (BMI), SAA, HGA, cholesterol, and CTH1. The outcome obtained, not only could help clinicians and researchers to monitor the trend of oxidative stress in an AKU-affected individual, but also could be used as a model for other research groups for improving the AKU-knowledge network.

### Prediction of QoL Scores Based on Clinical AKU Patients’ Clinical Data to Perform Patients’ Stratification

Patients’ stratification is one of the main goals that computational modelling together with databases can achieve. To achieve a first patients stratification in ApreciseKUre, a K-nearest neighbors algorithm (k-NN) was implemented to predict QoL scores starting from selected clinical biomarkers (Spiga et al., 2020 **)**. The innovative finding of this work is that, for the first time, we have found an ensemble of multiple complementary biomarkers whose combination produces better k-NN prediction of QoL scores than any single one. Moreover, due to the limited number of data available in a rare disease, it is essential to develop methods that would cope with the limited data size. The model has been therefore validated using surrogate data, because small dataset conditions and the associated random effects make validation of ML models for regression tasks impractical. Conventional methods, such as cross-validation, may become unreliable when the number of independent test samples is limited. The surrogate data method consists in the generation of a so-called “surrogate dataset” generated from random numbers and able to mimic the distribution of the original dataset in terms of their mean, standard deviation and range but they do not maintain the complex relationships between the variables of the real dataset.

Therefore, real-data models are consistent if they perform significantly better than the surrogate data models. In conclusion, this framework allowed ML algorithms to successfully predict clinical and QoL scores outcomes despite small datasets. The prediction of QoL score leads to a patient stratification making it addressable to several open issues in AKU with a strong clinical impact on early diagnosis, prediction of disease and of treatment outcome.

### A Tool Able to Investigate the Most Suitable Treatment in Accordance with AKU Patients’ QoL Scores

It has been already studied that QoL scores could identify health needs and to evaluate the impact of disease.

QoL of AKU patients was assessed through the following validated questionnaires (Braconi et al., 2010):

• Knee injury and Osteoarthritis Outcome Score (KOOS) (Roos and Lohmander, 2003), evaluating both short- and long-term consequences of knee injury. It contains 5 subscales: pain, other symptoms, function in daily living, function in sport and recreation, and knee-related QoL. Scores are normalized to a “0–100” scale, from extreme knee problems to no knee problems.
• Health Assessment Questionnaire (HAQ), including a disability index (haqDI) and a global pain visual analog scale (hapVAS). Scores are normalized to a “0–3” scale, from no difficulties to extreme ones.
• AKUSSI, incorporating clinically meaningful AKU outcomes combined with medical photography imaging investigations, and detailed questionnaires into a single score.

In this study (Spiga et al., 2021a), starting from the idea that there is a correlation between QoL and the clinical data deposited in the ApreciseKUre database, we have developed a ML model that performs a prediction of the QoL scores based on both personal, biochemical and clinical patients data. In this analysis, we considered the following QoL scores: AKUSSI joint pain, AKUSSI spinal pain, KOOS pain, KOOS symptoms, KOOS daily living, KOOS sport, KOOS QOL, HAQ-DI and hapVAS. All these QoL scores were standardized into three categorical variables (0, 1 and 2) corresponding to decreasing severity of health conditions (i.e., 0 is the worst condition and 2 is the best condition), to face the problem of data scarcity.

The classification was carried out using the Random Forest algorithm which suggests that KOOS indicator could be a useful factor to better understand symptoms and difficulties experienced by AKU patients (Spiga et al., 2020). KOOS prediction could be fundamental to assess consequences of primary osteoarthritis (OA), to identify the main important prognostic biomarkers, to help the clarification of physio-pathological mechanisms of AKU and ochronosis, and to assess the efficacy of future pharmacological treatments. Similarly, to most rare genetic diseases, the existing state-of-the-art treatment for AKU is unsatisfactory. With the only exception of Nitisinone, that resulted in reducing urinary excretion of HGA, in decreasing ochronosis and in improving clinical signs with a slower disease progression, there is still no other licensed therapy (Ranganath et al., 2020). Symptomatic treatments with anti inflammatories and painkillers are generally taken by AKU patients. The idea of personalizing the treatment according to “personal” and pathological features, as well as to special conditions could be the right approach to follow. For that reason, it has been looked for a correlation between the values of the QoL scores and the drugs the patients take. Fisher’s exact test was applied on all the combinations QoL score vs drug, employing the Benjamini–Hochberg procedure to deal with multiple comparisons. Antiarrhythmic and antihypertensive agents, as well as anti-inflammatories and opioid, resulted to be particularly effective in reducing AKU pain as suggested by a high correlation with KOOS scores, HAQ-DI, hap-VAS. Also, common drugs not related to specific AKU symptoms, such as cholesterol lowering and proton pomp inhibitors, showed a correlation with some QoL scores. In conclusion, vitamins resulted to be effective in the only case of KOOS pain evaluation.

### Comparison of Different Algorithms to Explore the Phenotype–Genotype Relationship

In order to obtain a first genotype/phenotype stratification of patients with AKU, our contribution (Spiga et al., 2021b) started from a preliminary statistical analysis based on Pearson’s correlation coefficient to evaluate the relationship between pairs of clinical data, biochemical parameters, and QoL scores. This correlation showed that biomarkers of chronic inflammation and amyloidosis, such as CHIT1 and SAA, did not strongly correlate with disease severity. In contrast, PTI showed a correlation with KOOS scores and age. Then, a stratification of patients into subgroups was performed using both K-means and Hierarchical Clustering. Three different stratification sizes (2, 3 and 4) were considered and the resulting clusters were grouped according to disease severity. Cluster assessment was performed by applying the nonparametric Kruskall-Wallis test. In addition, we calculated the Silhouette Score in order to test for consistency within items that were assigned to the same cluster. Finally, the distribution of HGD mutations in the obtained clusters was evaluated, with particular attention to the G161R, M368V, and A122 V mutations. G161R mutation, responsible for a dramatic reduction of HGD activity (Rodríguez et al., 2000), occurred in higher percentage in the most phenotypically severe clusters, while M368 and A122 V mutations, in which enzymatic activity of HGD is conserved for more than 30% (Rodríguez et al., 2000), occurred in higher percentage in less severe phenotypic sub-groups.

### AKUImg

Starting from the assumption that bio-imaging technologies are increasingly impacting on life sciences and sharing of image data is required to enable innovative future research, an ApreciseKUre plugin, called AKUImg (Rossi et al., 2020), was created. AKUImg is the first AKU-dedicated image repository. It is dedicated to the storage and analysis of AKU histopathological slides where images can be shared among registered researchers and clinicians to extend the AKU knowledge network. It allows to extend the recognition and reading of slides in the scientific community for an ultra-rare disease, like AKU by supporting clinicians and researchers with a user-friendly online tool able to distinguish between AKU or control cartilage slides. As a matter of fact, the plugin is also integrated with an accurate predictive model based on a standard image processing approach, namely histogram comparison, able to discriminate the presence of AKU by comparing histopathological images. Deep learning (DL) and convolutional neural networks (CNNs) have shown impressive results in many image-processing tasks. However, despite their popularity, they generally require huge datasets to reach good performance. Although we could divide each acquired image in patches, our dataset was not that big. To overcome the obstacle of the paucity of images available, the model we created has been a simple but effective binary classification of the knee cartilage. It performs a comparative analysis of the color histograms of the three channels revealing that AKU and healthy cartilages are easily distinguishable. Therefore, it has been calculated and stored color histograms for all the images in the dataset. For each new image to be classified, it has been evaluated the intersection region between the related histogram and all the histograms in the dataset. Finally, the test image has been assigned to the class with the largest intersection region. In conclusion, the algorithm can perform image classification with a high accuracy, making it a useful guide for non-AKU researchers and clinicians.

## Conclusion

Bioinformatics is an interdisciplinary field combining biology, computer science, information engineering, mathematics and statistics that develops methods and software tools to analyze and interpret biological data. Bioinformatics is taking a key role in big data analysis especially in healthcare, public health and in PM for a new understanding of the complexity of diseases and for tailoring the most appropriate treatment. PM is an innovative approach which aims to build a knowledge based network that can better guide individualized patient care, giving benefits in terms of health and quality of life. In this review, we focused on its application to an ultra-rare disease named AKU, characterized by no apparent genotype-phenotype relationship, no prognosis, and no therapy.

To develop an AKU-dedicated PME, clinical and experimental data have been collected and integrated in ApreciseKUre, a multi-purpose digital platform containing information of more than 200 AKU subjects, uniquely identified based on an anonymous key. Including updated case-data and samples from clinicians and patients, the researchers benefit from new information sources and can contribute to get a deeper knowledge of AKU.

However, ApreciseKUre is more than a data storage, as it also integrates computational predictive models able to map highly non-linear input and output and to investigate the health status of AKU patient patterns even when mechanistic relationships between model variables could not be determined. The main ML goal are listed below:

• Estimation of oxidative status trend of each AKU patient based on different biochemical predictors.
• Patients’ stratification based on QoL scores and clinical data
• Investigation of the most suitable treatment in accordance with AKU patients’ QoL scores
• Exploration of the phenotype–genotype relationships unknown in AKU

In conclusion, the application of computational algorithms together with the creation of digital databases will offer an opportunity to translate new data into actionable information. ApreciseKUre represents a guide applicable to other diseases, enabling data management, analysis and interpretation. Our sufficiently populated and standardized dataset allows for the achievement for the first time to extensively explore the phenotype-genotype distribution from a typical PM perspective.

## Abbreviations

AKU, Alkaptonuria; AKUSSI_jointpain, AKU Severity Score Index joint pain; AKUSSI_spinalpain, AKU Severity Score Index spinal pain; BMI, Body Mass Index; CatD, Cathepsin D; CysC, Cystatin C; CNN, convolutional neural networks; CTH1, Chitotriosidase; DL, Deep learning; GFR, glomerular filtration rate; HAQ-DI, Health Assessment Questionnaire Disability Index; HGA, Homogentisic Acid; HGD, Homogentisate 1,2-dioxigenase; hapVAS, Global pain visual analog scale; KOOSdaily_living, Knee injury and Osteoarthritis Outcome Score daily living; k-NN, K-nearest neighbors algorithm; KOOS_QOL, Knee injury and Osteoarthritis Outcome Score Quality of Life; KOOSpain, Knee injury and Osteoarthritis Outcome Score pain; KOOSsymptoms, Knee injury and Osteoarthritis Outcome Score symptoms; KOOSsport, Knee injury and Osteoarthritis Outcome Score sport; ML, machine learning; OA, osteoarthritis; PM, Precision Medicine; PME, Precision Medicine Ecosystem; PTI, Protein Thiolation Index; QoL, Quality of Life scores; SAA, Serum Amyloid A.
